# Evaluation of Paxillin Expression in Odontogenic Cysts and Tumors

**DOI:** 10.30476/dentjods.2023.98174.2056

**Published:** 2024-06-01

**Authors:** Azadeh Andisheh-Tadbir, Tina Sadat Shid-Moosavi, Fateme Gharibpour, Sahar Arabizadeh

**Affiliations:** 1 Oral and Dental Disease Research Center, Dept. of Oral and Maxillofacial Pathology, School of Dentistry, Shiraz University of Medical Sciences, Shiraz, Iran; 2 Postgraduate Student, Dept. of Pediatric Dentistry, School of Dentistry, Shiraz University of Medical Sciences, Shiraz, Iran; 3 Dental Sciences Research Center, Dept. of Orthodontics, School of Dentistry, Guilan University of Medical Sciences, Rasht, Iran; 4 Dentist, Ohio, USA

**Keywords:** Dentigerous cyst, Solid ameloblastoma, Unicystic ameloblastoma, Odontogenic keratocyst, Paxillin

## Abstract

**Statement of the Problem:**

Paxillin (PXN) is one of the proteins involved in cell adhesion. PXN and integrins constitute a key site for the focal adhesion between the cell and extracellular matrix. Several studies have shown that PXN is a factor in tumor formation, progression, invasion, and metastasis.

**Purpose:**

This study evaluated PXN expression in four types of odontogenic lesions with different aggressive behaviors

**Materials and Method:**

In this retrospective cross-sectional study, PXN expression was immunohistochemically assessed in 68 paraffin-embedded tissue samples from patients with the confirmed diagnosis of four types of odontogenic lesions, including 14 dentigerous cysts (DC), 20 odontogenic keratocyst (OKC), 16 unicystic ameloblastoma, and 18 solid ameloblastoma. The PXN expression in these samples were scored based on the percentage and intensity of immunoreactivity, and compared among the groups by Chi-square test.

**Results:**

The PXN marker was detected in the cytoplasm of tumor cells (unicystic and solid ameloblastoma) and the epithelial layer of cysts (DC and OKC). The intensively stained marker of PXN was observed in 9 cases (64.3%) of the DC, 14 cases (70%) of OKC, 12 cases (75%) of unicystic ameloblastoma, and 13 cases (72.2%) of solid ameloblastoma. However, there was not statistical difference of PXN protein expression between DC and OKC (*p* Value = 0.51) and unicystic and solid ameloblastoma (*p* = 0.58), also the same was true for cysts and tumors (*p* = 0.37).

**Conclusion:**

The expression of PXN is not related to the biological behaviors of odontogenic lesions.

## Introduction

Odontogenic cyst is a unique lesion affecting oral and maxillofacial tissue. Odontogenic cysts are caused by an inflammatory or developmental pathogenic source associated with the epithelium of tooth-forming apparatus [ 1 ], and they are one of the main causes of jaw bone destruction [ 2 ]. Odontogenic tumors originate from periodontal apparatus and tooth-bearing tissues in the jaw that form neoplastic growths. In radiological examinations, odontogenic tumors are usually radiolucent and some of them have sclerotic borders similar to odontogenic cysts that make their differentiation confusing from cysts [ 3 ].

The dentigerous cyst (DC), with a developmental origin, is the second most common jaw cyst after radicular cyst. Almost all of the DCs surround the crown of an un-erupted tooth and they are attached to the tooth's cementoenamel junction [ 1 ]. Odontogenic keratocyst (OKC) is a distinct form of developmental odontogenic lesion with specific histopathological characteristics and clinical behavior. Being locally invasive and having frequent recurrence are two major features of this lesion [ 4 ]. Ameloblastoma is a benign odontogenic tumor that commonly appears in the jawbone. They are known by their low prevalence, distinct biologic behavior, local invasion, and high risk of recurrence. Ameloblastoma is further classified into solid/multicystic, extraosseous/ peripheral, desmoplastic, and unicystic ameloblastoma [ 5 ]. The solid ameloblastoma mainly has follicular and plexiform histopathological patterns and is highly aggressive [ 6 ]. Unlike solid ameloblastoma, the unicystic ameloblastoma has a unilocular radiolucent pattern and is less aggressive and less invasive [ 7 ].

Paxillin (PXN) is one of the components of intracellular adaptor proteins with the scaffolding role for providing intracellular linkage between the actin cytoskeleton and transmembrane adhesion protein of integrin that extracellularly attaches to matrix proteins [ 8 - 10 ]. Proteins localized at the focal adhesion site consist of focal adhesion kinase (FAK), PXN, integrin, and vinculin that collectively manage cell adhesion and migration [ 8 ]. PXN also plays an important role in the transduction of signals between extracellular and intracellular responses, which is triggered by the interaction between integrin with the extracellular matrix (ECM) [ 9 ]. It is also incorporated in embryonic development, wound healing, and tumor metastasis [ 10 ]. The major cause of cancer cell migration, invasion, and metastasis is loss of adhesion to the ECM [ 8 ], and PXN has a key role in the tumor formation, progression, invasion, and metastasis [ 10 ].

Sun *et al*. [ 11 ] found that PXN overexpression was significantly related to high-grade glioma. Likewise, they indicated that PXN plays as an oncogene in glioma progression and is associated with higher mortality in survival analysis. Shekhar and Angadi [ 12 ] studied the pattern of PXN expression in varying grades of carcinoma and concluded that the PXN might be involved in the progression and growth of oral squamous cell carcinoma. Tanaka *et al*. [ 13 ] identified PXN as a direct target of MIR-199-5P and MIR-199-3P, which are involved in cancer pathogenesis in head and neck squamous cell carcinoma (HNSCC). Therefore, the present study was conducted to compare the PXN expression between four types of odontogenic lesions with different aggressive behaviors, including DC, OKC, solid ameloblastoma, and unicystic ameloblastoma.

## Materials and Method

### Study Population

In this retrospective cross-sectional study, paraffin-embedded tissue samples of DC, OKC, and ameloblastoma (unicystic and solid), which had a confirmed diagnosis, were retrieved from the archive of the Oral Pathology Department of Shiraz Dental School from 1997 to 2018, comprising 14 DC, 20 OKC, 16 unicystic ameloblastoma, and 18 solid ameloblastoma. The samples with uncertain diagnosis and insufficient tissue and also DC and OKC samples with severe inflammation were not selected. The protocol of study was approved by the Research Ethics Committee of Shiraz University of Medical Sciences (IR.SUMS.REC.1396.S1019).

### Immunohistochemistry

The evaluation of PXN protein expression was performed using EnVision labeled immunohistochemistry method. As previously described [ 14 ], the samples were fixed in 10% formalin and embedded in paraffin blocks and then 4 µm sections were prepared. The sections were deparaffinized in xylene, rehydrated with a graded alcohol series, and then treated with 3% hydrogen peroxide in methanol to block endogenous peroxidase activity. After rinsing the sections with phosphate-buffered saline, then subjected to microwave antigen retrieval in sodium citrate buffer (0.01 mol/L, pH 6.0). Then, the monoclonal antibody against PXN (Rabbit, Abcam Corporation, ab 32084, USA) was added as the primary antibody, followed by the streptavidin-horseradish peroxidase complex. For binding visualization in the sections, 3, 3 diaminobenzidine (DAB) chromogen was used and counterstained with hematoxylin. HeLa cells (a line from cervical cancer cells) were used with and without primary antibody as positive and negative control groups, respectively.

The PXN protein expression was quantified by assaying and scoring both the percentage and the intensity of immunoreactivity. The following scoring was considered to define the percentage of cells with positive immunoreactivity in every microscopic field: 0= less than 25%, 1= 25‑50% (weak), 2= >50‑75% (moderate), and 3= >75-100% (strong). The staining intensity was also scored as: 0 negative, 1 weak, 2 moderate, and 3 strong staining. Furthermore, the total score (ranging from 0 to 9) was calculated by multiplying the score of positive cells percentage to the score of staining intensity. Then, the samples with total score of 6 or more were classified as high-PXN expression and those with total score of less than 6 were classified as low-PXN expression [ 14 ].

### Statistical analysis

The Chi-square test was used to analyze the between- group comparisons and also between odontogenic cysts and tumors and between aggressive and non-aggressive odontogenic lesions. The statistical analyses of data were carried out using the SPSS software package (version 21, SPSS Inc, Chicago, USA), and the statistical significance was set at *p* < 0.05.

## Results

**Figure 1:**
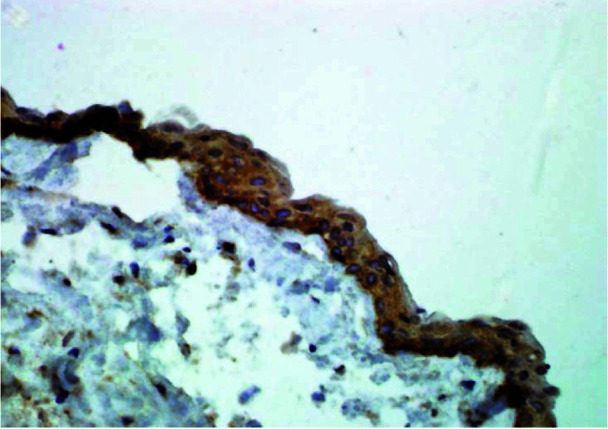
Representative microphotograph of immunohistochemical staining of cytoplasmic paxillin in dentigerous cyst (magnification 400×)

**Figure 2:**
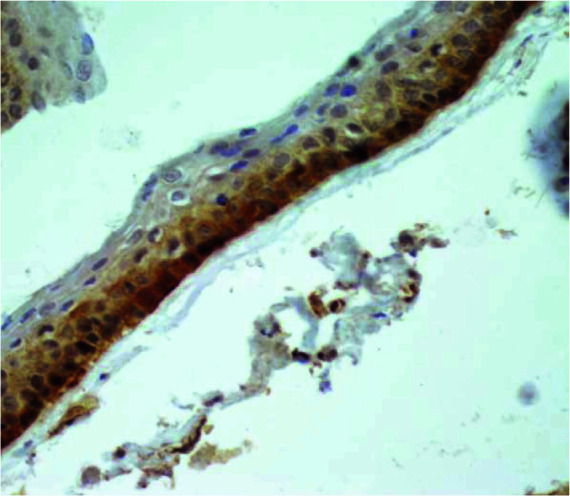
Representative microphotograph of immunohistochemical staining of cytoplasmic paxillin in odontogenic keratocyst (magnification 400×)

**Figure 3:**
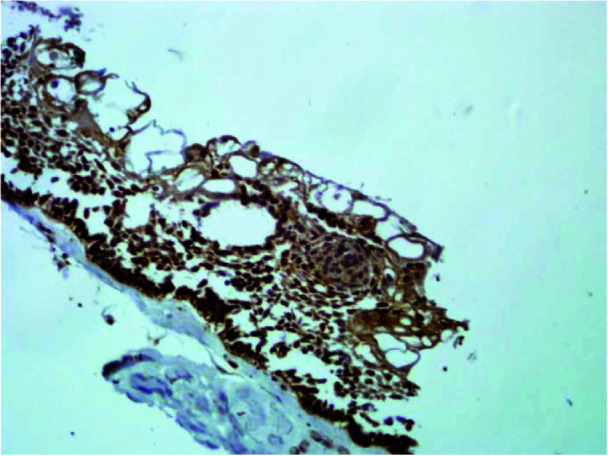
Representative microphotograph of immunohistochemical staining of cytoplasmic paxillin in unicystic ameloblastoma (magnification 200×)

**Figure 4:**
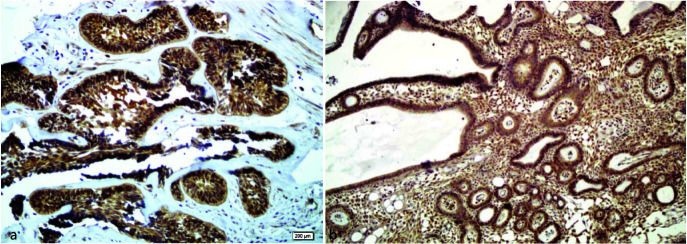
Representative microphotographs of immunohistochemical staining of cytoplasmic paxillin in **a:** Follicular solid ameloblastoma (magnification 200×) and **b:** Plexiform solid ameloblastoma (magnification 100×)

In this study, PXN staining was positively detected in epithelial lining of the cyst and cytoplasm of tumoral cells and in all layers of epithelial lining of DC (Figure 1), and the basal and parabasal layers of lining in OKC (Figure 2). In unicystic ameloblastoma (Figure 3) and solid ameloblastoma (Figure 4), PXN staining was seen in both ameloblast-like and stellate reticulum-like cells.

The obtained results for PXN total score, by multiplying scores of percentage and intensity of PXN immunoreactivity to each other, showed that there was high-PXN expression in 9 of 14 cases with DC (64.3%), 14 of 20 cases with OKC (70%), 12 of 16 cases with unicystic ameloblastoma (75%), and 13 of 18 cases with solid ameloblastoma (72.2%) (Table 1). However, the statistical analysis by Chi-square test did not show any significant difference in the PXN protein expression between DC and OKC (*p*= 0.51), unicystic ameloblastoma and solid ameloblastoma (*p*= 0.58), DC and unicystic ameloblastoma (*p* = 0.40), DC and solid ameloblastoma (*p*= 0.46), OKC and unicystic ameloblastoma (*p*= 0.52), and OKC and solid ameloblastoma (*p*= 0.58), as well as between odontogenic cysts and tumors (*p*= 0.37).

**Table 1:** Expression of paxillin in odontogenic cysts and tumors

Types of lesions		Low-paxillin expression	High-paxillin expression	*p* Value	
		Number (%)	Number (%)		
Cysts	Dentigerous Cyst	5 (35.7)	9 (64.3)	0.51	0.37
	Odontogenic Keratocyst	6 (30.0)	14 (70.0)		
Tumors	Unicystic Ameloblastoma	4 (25.0)	12 (75.0)	0.58	
	Solid Ameloblastoma	5 (27.8)	13 (72.2)		

## Discussion

In the present study, the PXN protein expression was immunohistochemically explored in four types of odontogenic lesions with different aggressive behaviors, including DC, OKC, solid ameloblastoma, and unicystic ameloblastoma, to find out the relationship between expression levels of PXN in them and their biological behaviors. 

It has been shown that PXN is involved in several types of cancers and strong PXN expression causes accelerated tumor cell proliferation, invasion, migration, and aggressiveness [ 8 , 15 - 18 ]. Liu *et al*. [ 10 ] demonstrated a strong association between PXN and invasiveness and metastases of gallbladder squamous cell/ adenosquamous carcinoma and adenocarcinoma and concluded that PXN overexpression has a significant role in clinical behavior and poor prognosis of these tumors. In a recent *in vitro* study, Song *et al*. [ 8 ] also investigated the abnormal expression of FAK and PXN and their correlation with the invasion and metastasis in oral squamous cell carcinoma (OSCC) cell line SCC-5. In the cultured SCC-5, they found a strong association of PXN expression with the increased chemoresistance, proliferation rate, migration, and invasion potential of side population (SP) cells, which had been previously shown to be responsible for tumor relapse. In contrast, Kandelman *et al*. [ 19 ] in a study on specimens collected by biopsy from women with non-nodular breast lesions could not find any relationship of PXN expression with invasion or prognostic variables and progression to malignancy. In another study, it was also demonstrated that PXN expression was not correlated with the invasiveness and tumor grade in breast carcinomas, although it was involved in the malignant transformation of breast epithelium [ 20 ].

In the present study, we did not identify any difference in expression of PXN between DC, OKC, solid ameloblastoma, and unicystic ameloblastoma, which have different aggressive behaviors. Bello *et al*. [ 21 ] immunohistochemically evaluated PXN and FAK in solid, unicystic and peripheral ameloblastoma and also adenomatoid odontogenic tumor (AOT), as a relatively non-aggressive and slow-growing tumor, and observed similar pattern of PXN expression in all tumor types. However, the expression of FAK was weak in all AOT cases and variable in ameloblastoma cases (mostly strong to weak). Therefore, they suggested the expression difference of FAK, but not PXN, might be involved in the patterns of biological behavior of these tumors. Baz and Mohamed [ 22 ] also observed the greatest cytoplasmic immunoreactivity for FAK in the aggressive lesions of the calcifying epithelial odontogenic tumor, conventional solid ameloblastoma and glandular odontogenic cyst but the lowest immunoreactivity for it in the less-aggressive unicystic ameloblastoma. In another study [ 14 ], we observed the higher PXN expression in paraffin-embedded tissue samples of salivary gland tumors, including malignant mucoepidermoid carcinoma, adenoid cystic carcinoma, and benign pleomorphic adenoma, in comparison to normal salivary gland. However, the expression of PXN in benign and malignant tumors was similar, and we did not find any correlation with clinicopathological features of patients [ 14 ].

The lack of difference between PXN expression and the patterns of biological behavior of odontogenic lesions suggests that the over-expression of PXN may occur in carcinomas and not in odontogenic cysts and tumors. Lin *et al*. [ 23 ] showed that PXN is an internal part of invadopodia, a finger-like protrusion used by cancerous cells to invade ECM and degrade it, and it is essential for the settlement of actin filaments in the invadopodia. Epithelial cells of benign odontogenic lesions do not use invadopodia for invasion and it can be the reason for no effectiveness of PXN in the local invasion of these lesions. Moreover, odontogenic lesions investigated in the present study were in the bone, there is epithelial barrier against their migration, and they never present metastasis. Hence, it can be assumed that PXN plays more role in cellular migration and invasion, as observed in carcinoma, rather than the local invasion.

In summary, the results of this study indicate that PXN is most likely not a key factor in determining the biological behaviors of DC, OKC, and ameloblastomas (solid and unicystic). Hence, it seems that targeting PXN has no clinical importance for treatment and lessening the destruction and extension of surgeries in invasive odontogenic cysts and tumors. We suggest further studies with larger samples size and other odontogenic cysts and tumors are necessary for identifying the accurate association between PXN and the biological behavior of odontogenic lesions.

## Conclusion

It was observed that the levels of PXN expression were not different among the four types of odontogenic lesions with different aggressive behaviors consists of DC, OKC, solid ameloblastoma, and unicystic ameloblastoma. Therefore, it can be concluded that the expression of PXN is not related to the patterns of biological behavior in odontogenic lesions.
